# Case Report: Borderline type I/II cryoglobulinemia associated with marginal zone lymphoma: a diagnostic challenge

**DOI:** 10.3389/fonc.2026.1838107

**Published:** 2026-05-19

**Authors:** Fabiana Crispo, Giovanna Mansueto, Francesca Pia Scioscia, Massimo Dante Di Somma, Diego Petrilli, Biagina Campisi, Fiorella D’Auria, Giulia Anna Carmen Vita, Giovanni D’Arena, Gabriella Bianchino

**Affiliations:** 1 Laboratory of Pre-Clinical and Translational Research, IRCCS-CROB Referral Cancer Center of Basilicata, Rionero in Vulture, PZ, Italy; 2 Hematology and Stem Cell Transplantation Unit, IRCCS-CROB Referral Cancer Center of Basilicata, Rionero in Vulture, PZ, Italy; 3 Unit of Clinical Pathology, IRCCS-CROB Referral Cancer Center of Basilicata, Rionero in Vulture, PZ, Italy; 4 Anatomical Pathology Department, IRCCS-CROB Referral Cancer Center of Basilicata, Rionero in Vulture, PZ, Italy; 5 Unit of Immunohematology and Transfusional Medicine, “San Luca” Hospital, ASL Salerno, Vallo della Lucania, Italy

**Keywords:** borderline type I/II cryoglobulinemia, Brouet’s classification, cryoglobulinemia, marginal zone lymphoma, rituximab

## Abstract

Cryoglobulinemia comprises heterogeneous disorders caused by immunoglobulins that precipitate at low temperatures, leading to vascular occlusion and immune–mediated tissue injury. Type I disease is typically linked to monoclonal gammopathies, whereas mixed forms (Type II or III) are characterized by immune complex-mediated vasculitis, rheumatoid factor activity, and renal involvement. The immune complexes are generally composed of IgM rheumatoid factor and IgG. We describe a 67–year–old woman with marginal zone lymphoma who developed an unusual cryoglobulinemia phenotype combining monoclonal IgM–κ cryoglobulins with clinical features characteristic of mixed disease, including purpura, transient rheumatoid factor positivity, and renal impairment. Diagnosis was delayed due to initial false–negative cryoglobulin testing and atypical presentation. Despite rituximab therapy, subsequent R–CVP, and intensive plasmapheresis, the patient’s condition progressed, ultimately requiring bendamustine–rituximab, after which she deteriorated and died shortly after discharge. The exceptional nature of this case lies the concomitant clinical manifestation of Type I and Type II cryoglobulinemia, which complicated the diagnostic process and therapeutic management. This case underscores a significant diagnostic challenge in hematology.

## Introduction

Cryoglobulinemia (CG) is a rare disease characterized by the precipitation of circulating immunoglobulins (Ig), known as cryoglobulins, in the plasma or serum at temperatures below 37 °C and their dissolution upon rewarming. Endothelial injury and end-organ damage may result from Ig complexes depositing in medium- and small-sized blood vessels throughout the body. With multiple distinct etiopathogenic pathways involved in organ lesions, cryoglobulinemia can be regarded as a heterogeneous disease with a broad variety of causes, symptoms, and evolution implications. This complicated scenario results in difficulties in timely diagnosis, as does the similarly complicated therapeutic scenario (1, 2).

According to Brouet’s classification, based on the immunoglobulin isotype composing the immune complex, clinicians discriminate cryoglobulinemia in: i) Type I, where immune precipitates are made up by single monoclonal Ig (most commonly IgM, rarely IgG or IgA); ii) Type II, where cryoglobulins are mixed due to the formation of polyclonal IgG and monoclonal IgM with rheumatoid factor activity (usually IgMκ plus IgGκ or IgGλ); iii) Type III, where the mixed immune complexes contain both polyclonal IgG and IgM aggregates (1). Hepatitis-C virus (HCV) infections, autoimmune diseases, primarily Sjögren syndrome, systemic lupus erythematosus, or rheumatoid arthritis, and occasionally B-cell lymphoproliferative disorders are frequently linked to Type II and Type III cryoglobulinemia. In contrast, Type I cryoglobulinemia is less prevalent, with a 5% incidence among patients with CG, and is always associated with B cell lymphoproliferative disorders, such as lymphomas and multiple myeloma (1, 3).

Despite sharing the same name, Type I and mixed cryoglobulinemia are two separate disorders with distinct clinical symptoms and disease courses due to different etiologies. Multiple thromboses of small and medium-sized vessels are typically caused by circulating Type I monoclonal cryoglobulins, and occasionally vascular inflammation accomplishes these clinical signals. In the Type I form, skin manifestations are largely reliant on ambient temperature, while cutaneous necrosis is more prominent and arthralgias are less common than in the mixed forms. Furthermore, the presence of large amounts of cryoglobulins, which are frequently observed in monoclonal cryoglobulinemia, causes hyperviscosity syndrome. In contrast, mixed cryoglobulinemia manifests as vascular purpura at the skin level, with lesions that are sometimes necrotic, and renal involvement is frequent and typically takes the form of membranoproliferative glomerulonephritis. Other typical signs of mixed cryoglobulinemia include small vessel vasculitis and inflammatory, bilateral, symmetric, and non-destructive arthralgias (2, 4).

Conventional treatment options for cryoglobulinemia include immunosuppressive pharmacological therapies (predominantly glucocorticoids and rituximab), plasmapheresis, and management of associated conditions. The choice depends on the underlying primary disorder, severity, and nature of organ involvement (5).

The laboratory procedures for the diagnosis of cryoglobulinemia are unsophisticated and handy, but operative conditions must be observed rigorously to avoid false-negative results due to cryoprecipitation, particularly in the early stages of the test. From the time of sampling until centrifugation, the physiological temperature must be maintained in accordance with the stringent preanalytical methodology for blood sample management. Notably, 9% of patients with cryoglobulinemia receive a delayed diagnosis due to initially false-negative testing (6), and therapeutic plans are frequently started too late to restrict clinical manifestations, the severity of which does not always correlate with cryoglobulin serum concentrations (4).

**Figure 1:**
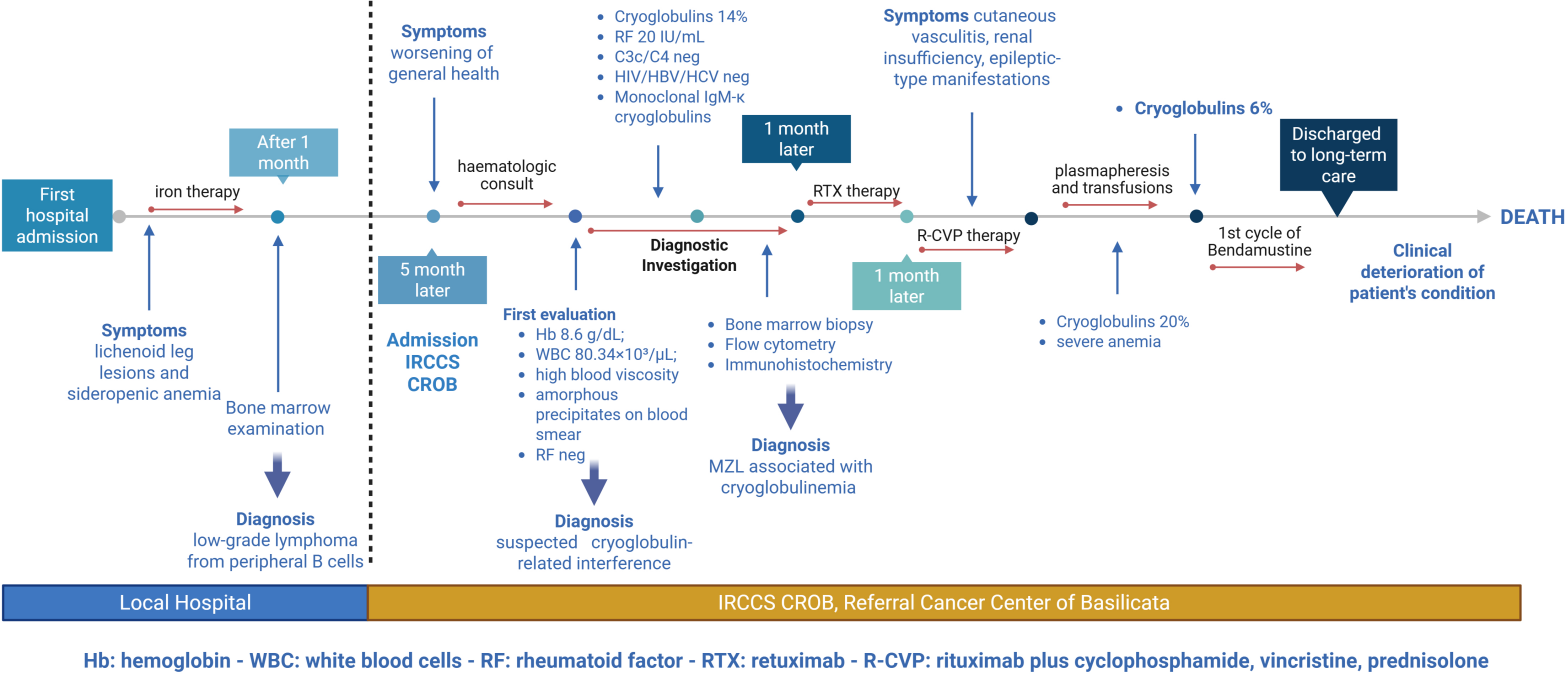
Clinical timeline summarizes the chronological progression of patient’s care and disease evolution.

This case report describes a rare presentation of marginal B-cell lymphoma in which the diagnosis of Type I cryoglobulinemia was established. The uniqueness of this case lies in the simultaneous manifestation of features classically associated with Type I cryoglobulinemia — namely monoclonality — alongside clinical symptoms typically seen in mixed cryoglobulinemia, including skin-level vascular purpura and transitional positivity to rheumatoid factor and renal involvement. The diagnosis and treatment process of this case is reflected in the form of a clinical timeline (**Figure 1**).

Recognizing such an atypical clinical profile is crucial, as this report underscores the importance of early identification and the timely initiation of appropriate therapy to optimize patient outcomes and prevent the progression of cryoglobulinemia related organ damage.

## Case presentation

A 67-year-old female, with a history of surgical removal of breast and parathyroid benign nodules was admitted to the Hematologic Day-Hospital Unit (DH) of IRCCS CROB, complaining pain of legs characterized by cutaneous dyschromia, ulcerated and scabbed lesions, and without a clear diagnosis from the hospital that took care of her previously.

Before admission to our Institute, the patient was treated at another local hospital, where she reported the appearance of leg lesions after being bitten by a tick. Contextually, she began to manifest sideropenic anemia, which was treated by the first hospital’s clinicians with iron therapy, first with oral supplementation and later with intravenous preparation. Due to the persistence of cutaneous lichenoid lesions and anemia, the patient underwent bone marrow and skin biopsies. Bone marrow examination highlighted the presence of a 30% lymphoid population of lymphocytes with the following immunophenotype: CD20+, CD5–, CD23+, Cyclin D1, and CD10–. However, skin biopsy excluded cutaneous involvement. The final diagnosis at the first hospital was infiltration of the bone marrow by low-grade lymphoma from peripheral B cells. No therapeutic protocol was prescribed to the patient after discharge.

Five months after a partial diagnosis made at the local hospital, the worsening of the patient’s general health encouraged her to seek a hematological consultation at our Institute.

**Figure 2:**
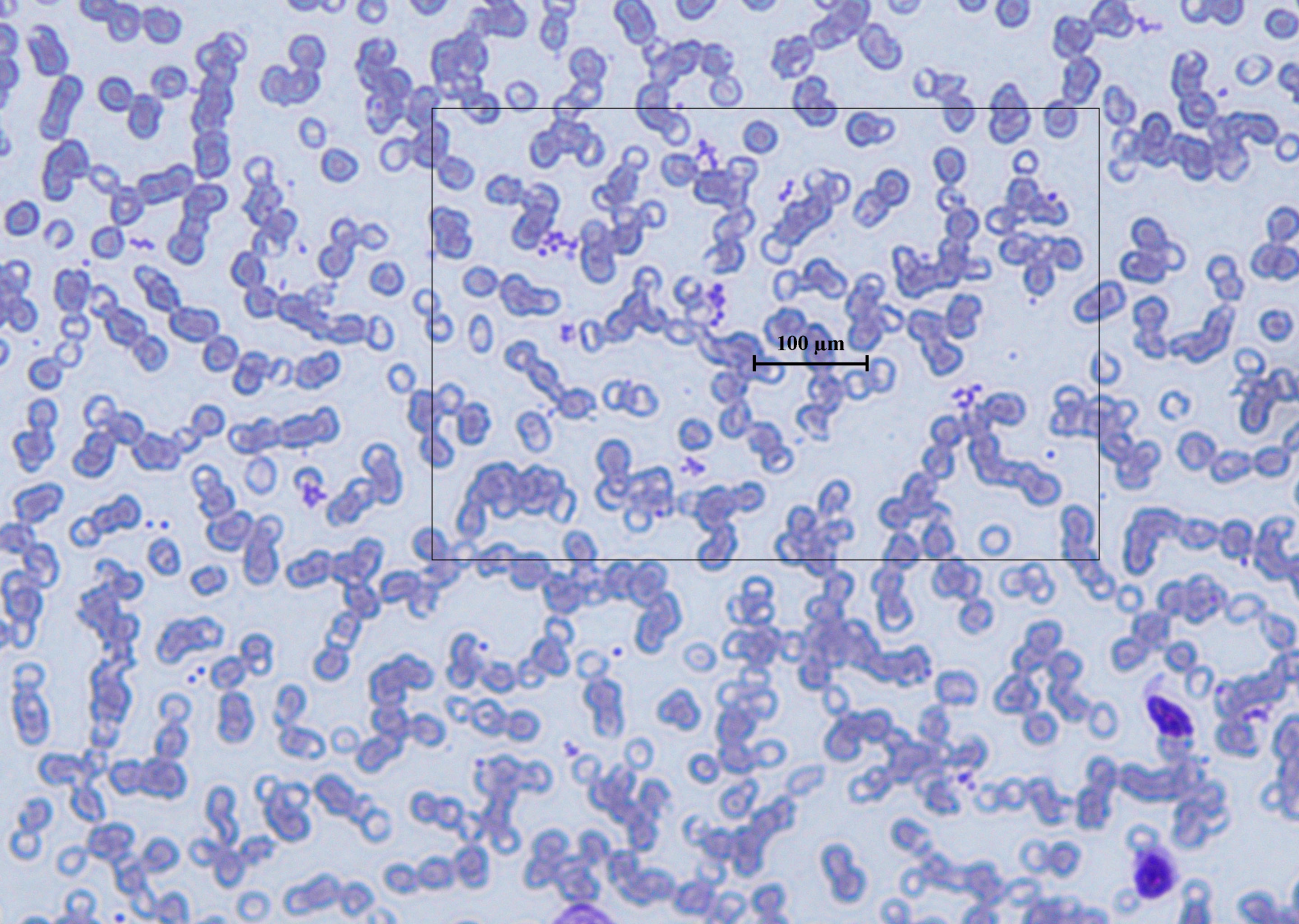
Peripheral blood smear showing erythrocyte rouleaux formation and platelet clumping, consistent with cryoglobulin-induced aggregation of red blood cells. Wright–Giemsa stain, original magnification ×40.

The first laboratory analysis revealed a complex scenario with notable anemia, demonstrated by low levels hemoglobin (Hb) 8.6 g/dL (12–18 g/dL) and transferrin 1.8 g/L (2.0-3.6 g/L), and a significant leucocytosis with WBC count 80.34×10^3^/uL (reference: 5.20-12.40×10^3^/uL) (neutrophils 45.9×10^3^/uL; lymphocytes 25.1×10^3^/uL; monocytes 8.50×10^3^/uL; eosinophils 0.3×10^3^/uL; basophils 0.4×10^3^/uL). The platelet count was 332.0×10^3^/uL (130-400×10^3^/uL), but high blood viscosity appeared after the sampling. However, the subsequent peripheral blood smear and cytofluorimetric analysis confirmed an important increment of leukocytes, suggesting a rheological interference in the hematocrit results, but mainly an amorphus pattern of precipitates dispersed among red blood cells (**Figure 2**).

Flow cytometric immunophenotype (**Table 1**) showed a marked predominance of T cells (CD3^+^ 90.8%, increased) with a balanced CD4^+^/CD8^+^ distribution (CD4^+^ 44.5%, CD8^+^ 44.6%). B cells were reduced (CD19^+^ 2.6%), and natural killer (NK) cells were within the upper limit of normal. Overall, this immunophenotypic pattern is consistent with relative T-cell expansion with B-cell lymphopenia, without evidence of a clonal T-cell population by surface markers. In the clinical context, these findings support an IgM monoclonal gammopathy–associated cryoglobulinemia rather than overt lymphoproliferative disease.

**Table 1:** Peripheral blood flow cytometric immunophenotyping demonstrates relative T-cell predominance with reduced B-cell population.

Immunophenotype	Lymphocytes (%)	Reference interval (%)
CD3^+^	90.8*	59-85
CD3^+^ CD4^+^	44.5	30-61
CD3^+^ CD8^+^	44.6	15-45
CD19^+^	2.6*	5-20
CD3^−^ CD16^+^ CD56^+^	6.2	4-28
CD3^−^ CD16^+^ CD56^+^	16.5*	2-16
Ratio CD4^+^/CD8^+^	1.0	0.8-2.4

Percentages of lymphocyte subsets are reported in comparison of reference intervals.

**Figure 3:**
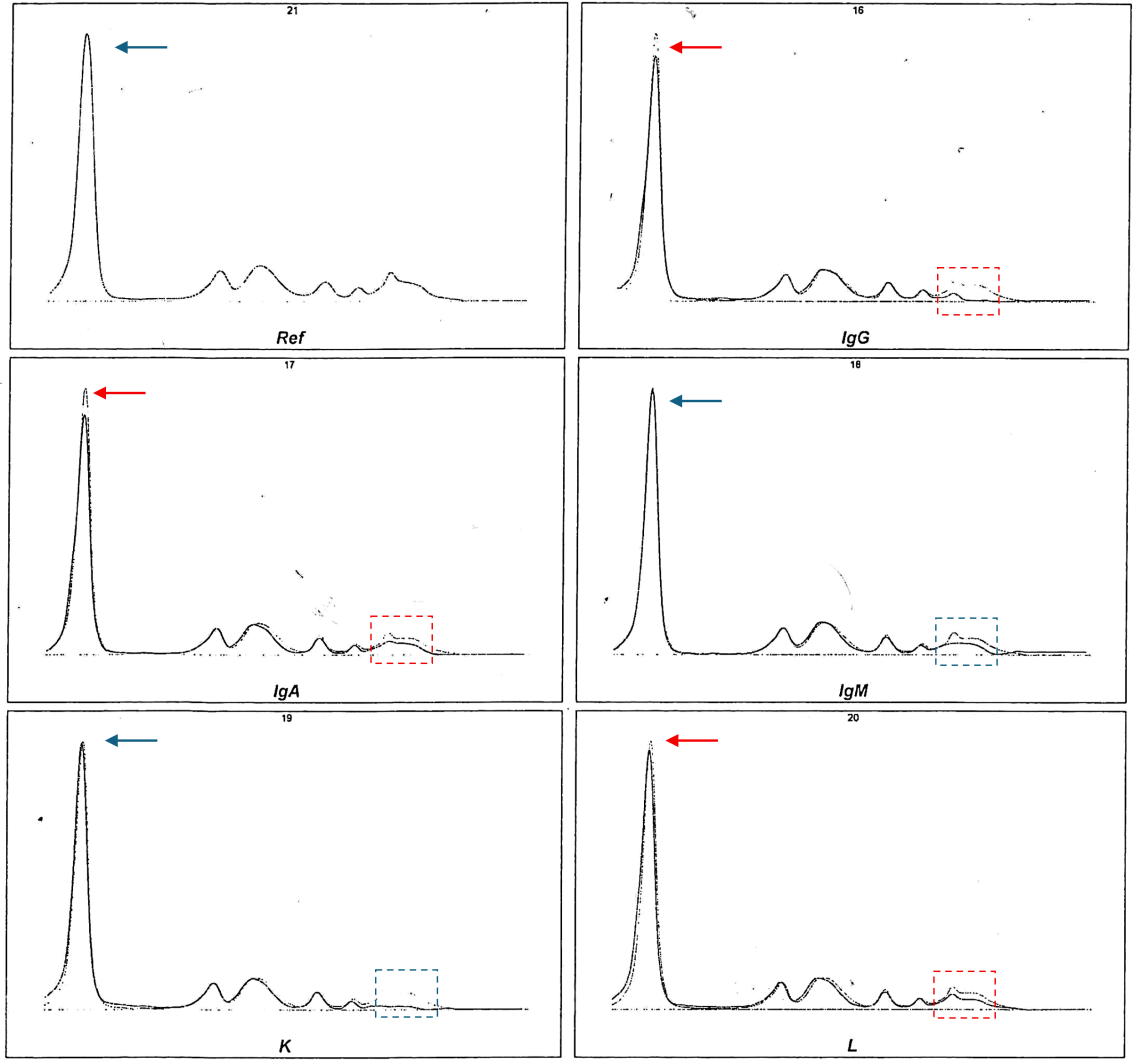
Capillary electrophoresis with immunosubtraction (CE/IS) performed on cryoprecipitate to identify the monoclonal immunoglobulin component. The six panels represent: Ref (reference pattern before subtraction), IgG (after IgG subtraction), IgA (after IgA subtraction), IgM (after IgM subtraction), κ (after κ light-chain subtraction), and λ (after λ light-chain subtraction). In each panel, the darker line represents the electrophoretic trace after subtraction, while the lighter line represents the profile before immunosubtraction. Red arrows, in IgG, IgA, and λ panels, highlight a minimal difference between the original and subtracted profiles of cryoprecipitate, including in the immunoglobulin region (red box). In contrast, a clear reduction of the monoclonal peak is observed after IgM and κ subtraction (blue box), with no changes in albumin peak (blue arrow), consistent with the presence of an IgM-κ monoclonal protein.

Further laboratory analysis, performed while maintaining the physiological temperature from sampling to centrifugation, revealed the presence of 14% cryoglobulins with positivity to rheumatoid factor 20 UI/mL (0–14 UI/mL) and negativity to complement factors C3c and C4. To classify cryoglobulinemia according to Brouet’s classification, cryoglobulins were evaluated by serum capillary electrophoresis/immunosubtraction, which revealed the monoclonal IgM κ light-chain nature of cryoprecipitates (**Figure 3**). The results of the serologic testing for viral infections, including the human immunodeficiency virus (HIV), hepatitis B virus (HBV), and chronic hepatitis C virus (HCV), were negative.

**Figure 4:**
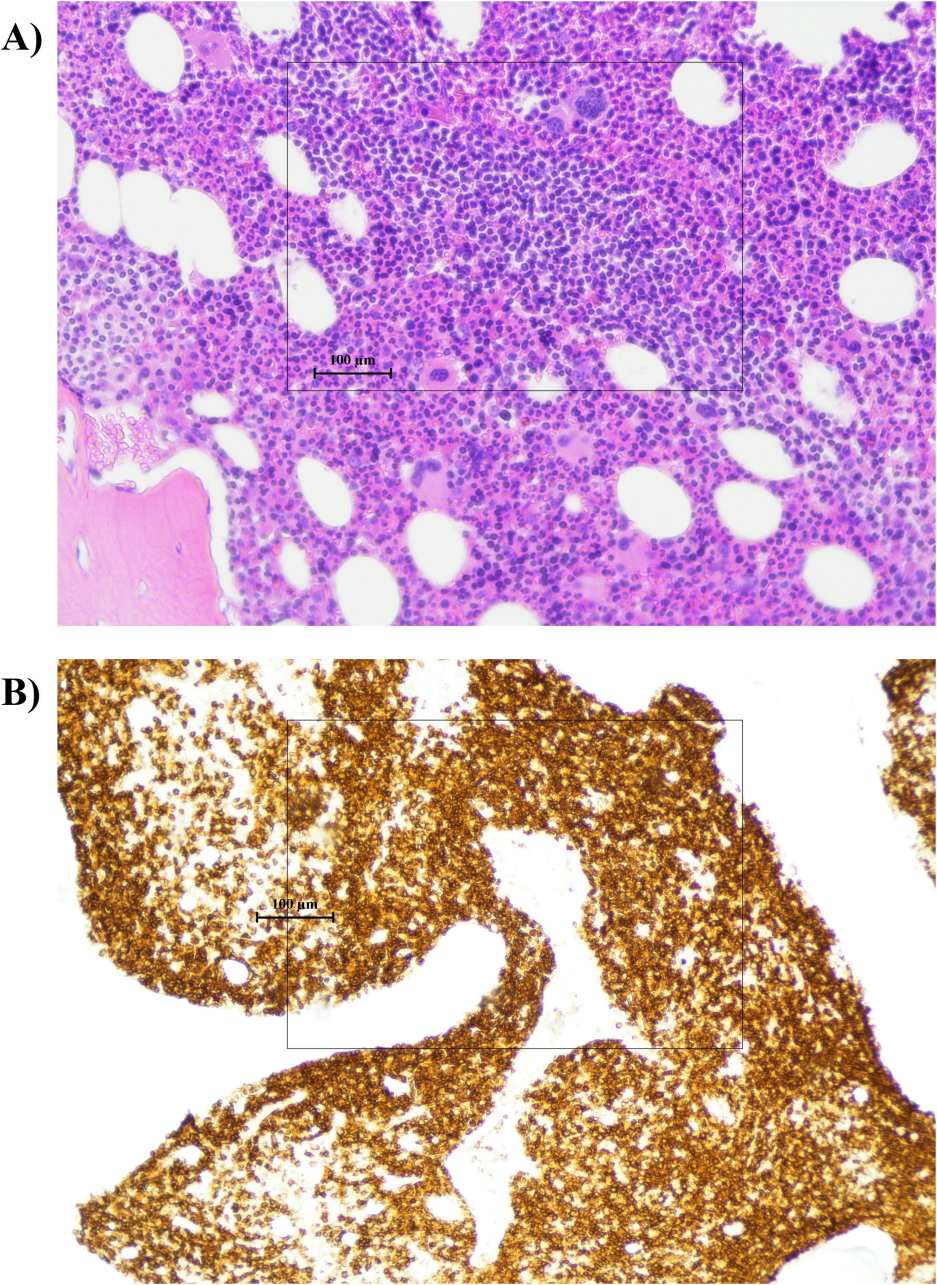
Bone marrow biopsy showing morphologic and immunophenotypic evidence of marginal zone lymphoma with focal–diffuse interstitial and paratrabecular infiltration: **(A)** hematoxylin and eosin (H&E) staining demonstrating diffuse infiltration by small B lymphocytes (original magnification 10×); **(B)** immunohistochemical staining for CD20 demonstrating strong positivity in the neoplastic B-cell population, highlighting the extent and distribution of marrow involvement (original magnification 10×).

Contextually, no palpable superficial lymphadenopathy was detected on physical examination. However, neck ultrasonography revealed the presence of small (subcentimeter) bilateral submandibular and laterocervical lymph nodes, which were not clinically appreciable, but need to be investigated further. A new bone marrow biopsy revealed that approximately 40% of infiltrated showed a mixed pattern of small lymphoid cells. The infiltrate was distributed both interstitially and in paratrabecular areas, with a combination of focal and diffuse involvement. The lymphoid cells were characterized by few cytoplasm, but regular nucleus profile, with the following immunophenotype: CD45^+^/LCA^+^, CD20^+^, CD79a^+^, BCL-2^+^, CD5^–^, CD23^–^, Cyclin D1^–^, CD10^–^, CD3^–^ (**Figures 4A, B**). This phenotypic pattern is compatible with low-grade marginal zone lymphoma (MZL) derived from mature B cells (7). No molecular rearrangements were observed in BCL2.

The combination of bone marrow histology and flow cytometry on peripheral blood provided sufficient diagnostic evidence of a clear clinical picture of MZL associated with Type I cryoglobulinemia. Due to the patient’s poor general clinical condition, prompt treatment with rituximab was initiated at a dose of 375 mg/m^2^, adopting the therapeutic plan of one infusion/week for four consecutive weeks. The treatment was well tolerated, and no adverse effects were observed.

After one month, disease progression was observed as confirmed by the deterioration of the general health condition of the patient and the appearance of new symptoms such as cutaneous vasculitis, renal insufficiency, with an increase in creatinine and uremia, macroscopic hematuria, and epileptic-type manifestations. Due to the low response, a new therapeutic plan, R-CVP (rituximab plus cyclophosphamide, vincristine, and prednisolone), was initiated for lymphoma treatment.

In this instance, the continuous significant increase of cryoglobulin content (14% at the first medical examination vs. 20% after two months) led to the initiation of specific treatment for cryoglobulinemia. The patients underwent eleven treatments of plasmapheresis procedures, supported by multiple concentrated red blood cell transfusion because of the persistence of severe anemia. After one month, the cryocrit dropped to 6% and bendamustine—an efficient and controllable therapy approach for indolent non-Hodgkin lymphoma—replaced CVP to continue the lymphoma’s treatment (8). The patient was discharged on oral steroids (prednisone 25 mg daily), outpatient plasmapheresis (once per week), and a second treatment with R-bendamustine three weeks after dismissions. Unfortunately, the patient passed away in a long-term care structure, few weeks after final discharge due to unknown circumstances.

## Discussions and conclusions

Cryoglobulinemia syndrome is a systemic disease that can manifest in several different ways, potentially affecting any organ. This variability reflects the diverse underlying pathogenic mechanisms and the immunochemical composition of cryoglobulins, even if it is clear that tissue damage occurs through two main mechanisms: the aggregation of cryoglobulins in microcirculation and the immune complex mediated inflammation of blood vessels and surrounding tissue.

Although Type I and mixed cryoglobulinemia display some distinct features, and that may aid in their differentiation diagnosis, sometimes the clinical symptoms of two subtypes may overlap. In particular, Type III (polyclonal) cryoglobulinemia could evolve into Type II (mixed monoclonal/polyclonal), especially in the setting of persistent B-cell stimulation, leading to clonal expansion and resembling those clinical features typically observed in Type I disease. This condition may complicate the diagnostic classification and contribute to delayed diagnosis.

Mixed cryoglobulinemia is considered a predisposing condition for lymphoproliferative disorders and confers a higher risk in developing B-cell lymphoma in patients with chronic HCV infection, compared to the general population (9). Marginal zone lymphoma, lymphoplasmacytic lymphoma, and high-grade diffuse large B-cell lymphoma are the lymphomas most frequently associated with this condition (10).

The present case is noteworthy because it illustrates an atypical clinical presentation of Type I cryoglobulinemia associated with marginal zone lymphoma, in which features classically attributed to mixed cryoglobulinemia, such as vascular purpura, transient rheumatoid factor positivity, and renal involvement, were also observed. This overlap underscores the limitations of a rigid clinical distinction between cryoglobulinemia subtypes and highlights the dynamic nature of B-cell–driven immune dysregulation. The cryoglobulin-related manifestations in the MZL patient, caused by monoclonal IgM κ light-chain cryoprecipitates, included not only vascular occlusion and hyperviscosity syndrome, typical of Type I disease, but also immune-mediated inflammatory small-vessel vasculitis more characteristic of mixed cryoglobulinemia. This case further emphasizes the importance of meticulous preanalytical handling of blood samples for cryoglobulin detection, as delayed or false-negative results may significantly postpone diagnosis and treatment initiation, adversely affecting patient outcomes.

From a therapeutic perspective, management of cryoglobulinemia associated with lymphoproliferative disorders requires a dual approach targeting both the underlying hematologic malignancy and the cryoglobulin-mediated organ damage. In this patient, initial treatment with rituximab alone was insufficient to control disease progression, necessitating escalation to combined immunochemotherapy and adjunctive plasmapheresis. The progressive increase in cryocrit despite therapy highlights the aggressive clinical course and the difficulty of achieving disease control in such complex presentations.

An additional point to consider in the interpretation of this case is the possibility of histologic transformation of MZL into a more aggressive B-cell lymphoma. Although histologic transformation, is relatively uncommon in MZL, reported with a 5- and 10-year cumulative incidence of approximately 5-8% across all subtypes (11), it is a well-recognized event associated with rapid evolution to aggressive malignancy, worsening clinical condition and poor prognosis. Recent studies have highlighted the molecular mechanisms underlying transformation from indolent to aggressive B-cell malignancies, emphasizing the biological complexity of this process, its impact on outcomes and its cruciality for developing novel targeted treatments (12).

In the present case, a PET-guided lymph node biopsy was not performed, and the diagnosis relied on bone marrow findings. Consequently, histologic transformation cannot be formally excluded, and the aggressive clinical course observed could be consistent with such a scenario. However, due to the patient’s poor general condition, further invasive diagnostic procedures were excluded, limiting the interpretation of the clinical evolution of disease.

It was not clear if an early correct diagnosis of cryoglobulinemia and/or a more comprehensive interpretation of the MZL evolution could have been increased the life quality and expect for the patient, but it is evident that Type I cryoglobulinemia represents a very challenging disease with regard to therapeutic options, due to the hematologic disorder underlies the pathophysiological mechanism of cryoprecipitate formation.

Although Type II and Type III mixed cryoglobulinemia have promising therapeutic approaches directed to HCV infectious eradication combined with treatment targeting vascular inflammation, further efforts should be planned for increasing translational research and multicenter, randomized controlled trials focusing on noninfectious non-infectious mixed and Type I cryoglobulinemia, with the aim to improve patient outcomes and long-term prognosis.

## Data Availability

The original contributions presented in the study are included in the article/supplementary material. Further inquiries can be directed to the corresponding author/s.
